# PER2 expression and cellular localization play a critical role in tumor aggressiveness and drug resistance in an *in vitro* model of hepatocellular carcinoma

**DOI:** 10.20517/cdr.2024.193

**Published:** 2025-06-03

**Authors:** Mariarosaria Negri, Feliciana Amatrudo, Donatella Paola Provvisiero, Roberta Patalano, Giovanna Trinchese, Fabiano Cimmino, Cristina de Angelis, Chiara Simeoli, Renata Simona Auriemma, Maria Pina Mollica, Annamaria Colao, Rosario Pivonello, Claudia Pivonello

**Affiliations:** ^1^Department of Wellness, Nutrition and Sport, Pegaso Telematic University, Naples 80143, Italy.; ^2^Department of Clinical Medicine and Surgery, Section of Endocrinology, Diabetology, Andrology and Nutrition, Federico II University, Naples 80131, Italy.; ^3^Department of Biology, Federico II University, Naples 80126, Italy.; ^4^UNESCO Chair for Health Education and Sustainable Development, Federico II University, Naples 80131, Italy.; ^5^Department of Public Health, Federico II University, Naples 80131, Italy.; ^#^These authors have contributed equally to this work.

**Keywords:** Clock genes, Period 2, hepatocellular carcinoma, epithelial-mesenchymal transition (EMT), everolimus, sorafenib, drug resistance

## Abstract

**Aim:** The current in vitro study investigated the role of Period 2 (PER2) in aggressiveness and the acquisition of drug resistance in hepatocellular carcinoma (HCC).

**Methods:** Parental PLC/PRF/5 cells, along with everolimus-resistant (EveR) and Sorafenib-resistant (SorR) cell lines, were used in this study. PER2 expression was silenced using siRNA knockdown (KD) and blocked using CRISPR/Cas9 Plasmid knockout (KO). PER2 expression levels were assessed by quantitative real-time reverse transcription polymerase chain reaction and immunofluorescence, together with markers of epithelial-mesenchymal transition, casein kinase 1ε (CK1ε), and tumor protein p53. Modulation of p53, p21, cellular myelocytomatosis oncogene, and mouse double minute 2 homolog was investigated by western blot. Mitochondrial activity was evaluated using the Seahorse System. The role of PER2 on the onset of aggressiveness was examined through assays of cell proliferation, migration, and colony formation.

**Results:** PLC/PRF/5 everolimus-resistant (EveR), SorR, PER2 KD, and PER2 KO cells expressed significantly lower PER2 mRNA and protein levels compared to the parental PLC/PRF/5 cells. Remarkably, in PLC/PRF/5 EveR and SorR cells, PER2 protein was entirely localized in the cytoplasm, where it colocalized with CK1ε, in contrast to the parental cells. In PLC/PRF/5 EveR, PER2 KD and PER2 KO cells, but not in SorR cells, E-cadherin was significantly decreased while vimentin and ZEB1 protein levels were significantly increased across all modified cell models. Interestingly, p53 expression was reduced in PER2 KO cells and completely absent in PLC/PRF/5 EveR and SorR cells. Consistent with these findings, the inhibitory effect of everolimus (10^-9^ M) and sorafenib (5 × 10^-6^ M) on cell proliferation, migration, and colony formation observed in parental PLC/PRF/5 cells were reversed in PER2 KD and KO cells, which was accompanied by upregulation of oncogenes, downregulation of tumor suppressor genes, and alterations in mitochondrial activity.

**Conclusion:** These results suggest that the acquisition of an aggressive phenotype is characterized by reduced PER2 expression and loss of its nuclear translocation, which, in turn, is associated with resistance to systemic therapy in hepatocellular carcinoma.

## INTRODUCTION

The entire cohort of organs and tissues throughout the body encompasses peripheral circadian clocks, which are synchronized by the master clock located in the suprachiasmatic nucleus (SCN) through hormonal and neuronal factors. Both the master and peripheral clocks operate using the same set of clock genes, including BMAL1, CLOCK, PER1-3, and CRY1-2, which generate a cell-autonomous molecular oscillator. This oscillator is based on the auto-regulatory transcriptional-translational feedback loop mechanisms that sustain a periodicity of about 24 h^[1,2]^. Circadian molecular oscillators regulate several biological processes and, at the cellular level, are involved in the regulation of the cell cycle by controlling the timing of cell cycle progression, cell death and survival by modulating the expression of key elements of cell growth^[3]^. Clock genes have also been shown to affect pathological processes in cancer^[4]^, and genetic variants in clock genes have been closely associated with an increased risk of various cancers^[5,6]^, including hepatocellular carcinoma (HCC)^[7]^.

HCC represents the most frequent form of primary liver cancer worldwide^[8]^. In recent years, immunotherapy, including immune checkpoint inhibitors (ICIs), has revolutionized HCC treatment^[9]^. Currently, for the management of advanced-stage HCC [Barcelona clinic liver cancer (BCLC) stage C], the U.S. Food and Drug Administration (FDA) has approved the use of ICIs, including combination therapies^[10,11]^, as these confer a superior survival benefit compared to the tyrosine kinase inhibitor (TKI) sorafenib (SOR). Despite this evidence, 15%-20% of HCC patients, particularly those with underlying autoimmune liver disease such as autoimmune hepatitis, can benefit only from receiving TKIs such as SOR or lenvatinib^[12]^. Nevertheless, acquired resistance to targeted therapies frequently occurs, often due to the reactivation of signaling pathways initially inhibited by the TKI. This can result from alterations in upstream or downstream regulatory pathways or secondary modifications of the drug targets.

Besides its well-known activity as a clock gene, Period 2 (PER2) emerged as a tumor suppressor gene in various cancers^[4,13]^ including HCC^[7,14]^. Indeed, relative *Per2* expression was found to be reduced in HCC compared to the surrounding peritumoral tissue in mice models^[15]^. Similarly, both PER2 gene and protein expression levels were significantly lower in human HCC tissues compared with the paired cancer-adjacent tissues^[16-18]^. Interestingly, Chen *et al.* found that PER2 expression correlates with China liver cancer (CNLC) staging and immune cell infiltration, suggesting a role in regulating the tumor microenvironment^[16]^. Moreover, functional enrichment analysis of PER2-related differentially expressed genes revealed that PER2 may regulate mitochondrial oxidative phosphorylation, transcription and translation, amino acid metabolism, and other pathways, thereby promoting HCC proliferation, metastasis, and invasion^[16]^.

Nevertheless, the role of PER2 expression and modulation in drug resistance remains poorly understood in HCC, mainly due to the difficulty in obtaining tissue samples from patients with unresectable HCC in the advanced stage (BCLC stage C), for whom the treatment with TKIs is typically indicated. To overcome this limitation, an HCC cell model resistant to the mammalian target of rapamycin (mTOR) inhibitor everolimus (EVE) was developed through long-term *in vitro* drug exposure. This model demonstrated that chronic exposure to EVE leads to acquired drug resistance and aggressive phenotype, characterized by increased expression of mesenchymal markers^[19]^. The current study aimed to investigate the role of PER2 modulation in the acquisition of aggressiveness and drug resistance in human HCC cell models rendering resistant to EVE (EveR cells), as well as an additional model resistant to SOR spawned after long-term *in vitro* exposure, defined SOR resistant (SorR) cells.

## MATERIALS AND METHODS

### *In silico* analysis of PER2 gene expression in HCC cohorts and human HCC cell lines

*PER2* gene expression (RNAseq data) was evaluated based on The Cancer Genome Atlas (TCGA) datasets using The Gene Expression Profiling Interactive Analysis (GEPIA2 - http://gepia.cancer-pku.cn/) webserver in the HCC cohort of tumor samples (*n* = 369) and normal liver tissue samples (*n* = 50). *PER2* gene expression was reported as log_2_ transcripts per million (TPM) + 1. Moreover, *PER2* gene expression was also evaluated in various human HCC cell lines using Cancer Cell Line Encyclopedia (CCLE), analyzed by the webserver Depmap Portal (https://depmap.org/portal/interactive/) using a dataset posted in 2022 (Expression Public 22Q4). The potential prognostic role of *PER2* gene expression was evaluated by calculating the overall survival using the Kaplan-Meier method using multiple datasets in GEPIA2.

### Compounds, cell lines and treatments.

EVE and SOR were supplied by Selleck Chemicals (UK). EVE was resuspended as previously described^[19]^. SOR was dissolved in dimethyl sulfoxide (100%) and concentrated aliquots (10^-3^ M) were stored at -80 °C. Fresh aliquots were defrosted before each new experiment and used for serial dilutions.

The PLC/PRF/5 parental cell line was purchased by the American Type Culture Collection (ATCC) bank and cultured in Dulbecco’s modified eagle’s medium (DMEM) medium with 10% of Fetal Bovine Serum (FBS), 1 × 10^5^ U/L penicillin and streptomycin, 0.1% of fungizone (Gibco), and 1% of minimum essential medium (MEM) non-essential Amino Acids (100x) (ThermoFisher).

Everolimus-resistant (EveR) cell line was developed as previously reported^[19]^. SOR-resistant cell line (SorR) was developed by continuous treatment with SOR (7.5 × 10^-6^ M) for 12 months. The acquired cell resistance was established by performing cell proliferation analysis evaluated by DNA assay, after 6 days of treatment with escalating doses of SOR ranging from 10^-6^ to 10^-5^ M. For the experiments in which the effect of EVE and SOR were evaluated in cells in which *PER2* was knocked down and knocked out, 10^-9^ M EVE and 5 × 10^-6^ M SOR were used.

### RNA isolation and real-time reverse transcription polymerase chain reaction

Total RNA isolation and real-time reverse transcription polymerase chain reaction (RT-qPCR) were performed following the protocol previously described^[20]^.

The cDNA was used for the quantification of *PER2* mRNA levels. The total reaction volume (12.5 μL) consisted of 5 μL of cDNA, 7 μL of TaqMan® Universal PCR Mastermix (Applied Biosystems, Branchburg, NJ, USA), and 0.5 μL of primers-probes (Applied Biosystems, Branchburg, NJ, USA). Primers and probes assay ID were: *PER2* gene Hs00256143_ml and housekeeping *Hypoxanthine Phosphoribosyltransferase 1* (*HPRT)* Hs02800695_ml. RT-qPCR was performed using the standard protocol with StepOne Plus Real-Time PCR System. Briefly, after two initial heating steps at 50 °C (2 min) and 95 °C (10 min), samples were subjected to 40 cycles of denaturation at 95 °C (15 s) and annealing at 60 °C (60 s). All samples were assayed in duplicate. Values were normalized against the expression of the housekeeping gene *HPRT*. Results are expressed as the mean of three different experiments. The relative expression of target genes was calculated using the comparative threshold method, 2^-ΔCt^. Concomitantly to cDNA samples, reactions were also performed in the absence of a cDNA template to exclude contamination of the PCR mixtures.

### Protein extraction and Western blot analysis

Cell lysis and total protein extraction were performed as previously described^[21]^. The nuclear and cytoplasmic extraction was prepared using the NE-PER Nuclear Cytoplasmic Extraction Reagents kit (Thermo Scientific, code 78833) according to the manufacturer's instructions. Briefly, parental PLC/PRF/5, PLC/PRF/5 PER2 KD, PLC/PRF/5 PER2 KO, PLC/PRF/5 EveR, and PLC/PRF/5 SorR cells were washed twice with cold PBS (1X) and centrifuged at 500 x g for 3 min. The cell pellet was suspended in 200 μL of ice-cold cytoplasmic extraction reagent I (CER I) and vortexed. The suspension was incubated on ice for 10 min, followed by the addition of 11 μL of an ice-cold second cytoplasmic extraction reagent II (CER II), vortexed for 5 s, incubated on ice for 1 min, and centrifuged for 5 min at 16,000 x g. The supernatant fraction (cytoplasmic extract) was transferred to a pre-chilled tube. The insoluble pellet fraction, which contains crude nuclei, was resuspended in 100 μL of ice-cold nuclear extraction reagent (NER) and vortexed for 15 s every 10 min for a total of 40 min, then centrifuged for 10 min at 16,000 x g. The resulting supernatant, constituting the nuclear extract, was used for the Western blot (WB) analysis that was performed as previously described^[21]^. Primary antibodies specific for PER2 (Abcam, ab179813, rabbit monoclonal, dilution 1/1000), mouse double minute 2 homolog (MDM2) (Santa Cruz, sc-965, mouse monoclonal, dilution 1/500), p53 (Cell Signaling, #2524, mouse monoclonal, dilution 1/500), p21 (Invitrogen, GT-1032, mouse monoclonal, dilution 1/1,000), cellular myelocytomatosis oncogene (c-MYC) (Cell Signaling, #5605, rabbit monoclonal, dilution 1/1,000), *β*-actin (Sigma Aldrich, A4700, mouse monoclonal, dilution 1/3,000), and TATA-box-binding protein (Abcam, ab300656, mouse monoclonal, dilution 1/300) were probed on nitrocellulose filters overnight. Peroxidize-conjugated secondary antibodies (Donkey anti-Rabbit IgG, DkxRb-003-DHRPX, ImmunoReagents, Inc. or Goat anti-Mouse IgG, GtxMu-003-DHRPX, ImmunoReagents inc.) were probed for 1 h. The horseradish peroxidase substrate for enhanced chemiluminescence system detected immunoreactive bands, and the blot was exposed to ImageQuant Las 4000 (GE Healthcare).

### PER2 knockdown with siRNA transfection

To knock down *PER2* expression, small interfering RNAs (siRNAs) (TriFECTa Kit DsiRNA Duplex, IDT Integrated DNA Technologies) have been tested. Three different siRNA oligonucleotides were used according to the manufacturer’s instructions: hs.Ri.PER2.13.1 (sense: 5’-GAUGCCAAGCAUCAAGUCAUGUUTT-3’; antisense: 3’-CACUACGGUUCGUAGUUCAGUACAAAA-5’); hs.Ri.PER2.13.2 (sense: 5’-GCAUGAAUGGAUCGCGGAAUUUCC-3’; antisense: 5’- GUCGUACUUACCUAUGCGCCUUAAAGG-3’); and hs.Ri.PER2.13.3 (sense: 5’-AGUCAUUAUUCUCAUGAAUCUGGAG-3’; antisense: 3’-CUUCAGUAAUAAGAGUACUUAGACCUC-5’) (IDT Integrated DNA Technologies). Negative control siRNA (IDT Integrated DNA Technologies) was used as control.

Transfection was performed for 24 h using Lipofectamine 2000 (#11668019, Invitrogen, Carlsbad, CA, USA) following the manufacturer’s protocol, with siRNA at a final concentration of 10 nm. Briefly, 1.5 × 10^5^ PLC/PRF/5 cells were seeded in 35 mm Petri dish with 1.5 mL DMEM medium at 37 °C with 5% of CO_2_. When cells reached 70%-80% of confluence, 1.75 µL siRNA and 2.5 µL Lipofectamine 2,000 were homogeneously dissolved in 250 μL siRNA transfection buffer Opti-MEM (#11058021, ThermoFisher). The mix was left at room temperature for 15 min and then added to the culture medium. Finally, the cells were cultured for 24 h at 37 °C with 5% CO_2_. siRNA transfection efficiency was verified by WB analysis using PER2 antibody (GT5310, ThermoFisher, Italy). Immunofluorescence (IF) analysis was performed to confirm the knockdown (KD) of PER2 compared to parental cells using PER2 antibody (ab179813, Abcam).

### PER2 knockout with CRISPR/Cas9 technology

To knock out PER2 gene expression, Per2 CRISPR/CAS9 knockout (KO) Plasmid (sc-401089-KO-2, Santa Cruz Biotechonology, Inc.) was used. CRISPR/Cas9 transfection was performed on PLC/PRF/5 parental cells following the manufacturing instructions. Briefly, 2.5 × 10^5^ PLC/PRF/5 parental cells were cultured on six-well plates, with 3 mL of antibiotic-free standard growth DMEM medium, for 24 h before transfection. The cells were co-transfected with 2 µg of human PER2 KO plasmid (sc-401089-KO-2) and 2 µg of human PER2 HDR plasmid (sc-401089-HDR-2) using 10 µL of UltraCruz Transfection Reagent (sc-395739, Santa Cruz Biotechnology, Inc.) in a final volume of 150 µL Plasmid Transfection Medium (sc-108062, Santa Cruz Biotechnology, Inc.). This transfection assured a complete gene KO of PER2. Concomitantly, the HDR plasmid incorporated a puromycin resistance gene for the selection of cells where Cas9-induced DNA cleavage had occurred. Successful CRISPR/Cas9 KO plasmid transfection was confirmed using a medium containing 1 µg/mL Puromycin (sc-108071, Santa Cruz Biotechnology, Inc.) for 3 days. Control PLC/PRF/5 parental cells were created using control plasmid (sc-418922; Santa Cruz, Inc.) according to the manufacturing instructions.

To confirm complete PER2 allelic KO, IF analysis was performed comparing transfected cells to parental cells using PER2 antibody (ab179813, Abcam).

### IF staining

The parental PLC/PRF/5, PLC/PRF/5 PER2 KD, PLC/PRF/5 PER2 KO, PLC/PRF/5 EveR, and PLC/PRF/5 SorR cells were used for IF staining. For all cell models, 6 × 10^4^ cells were seeded in µ-Dish 35 mm (IBIDI) and were grown for 24 h. The PLC/PRF/5 parental cells were then transfected with siPER2 10 nm, using Lipofectamine 2,000 in the same condition described above.

IF staining was performed as previously described^[21]^. The cells were incubated with primary antibodies against vimentin (Abcam, ab92547, rabbit monoclonal, dilution 1/250), E-cadherin (Santa Cruz, sc-21791, mouse monoclonal, dilution 1/100), ZEB1 (Santa Cruz, sc-515797, mouse monoclonal, dilution 1/50), PER2 (Abcam, ab179813, rabbit monoclonal, dilution 1/50), p53 (Cell Signaling, #2524, rabbit monoclonal, dilution 1/250), and CK1ε (Abcam, ab302638, mouse recombinant monoclonal, dilution 1/100) for 1.5 h. Slides were then washed 3 times in 0.1% Triton/ phosphate-buffered saline for 5 min and incubated with the secondary antibodies for 1 h (Millipore, AP124F, goat anti-mouse, fluorescein isothiocyanate conjugated, dilution 1/500; ImmunoReagent, Gtx-Rb-003-DRHO, goat anti-rabbit, tetramethylrhodamine isothiocyanate conjugated, dilution 1/500; Goat anti-Mouse immunoglobulin G, Cod. A-11001, (heavy and light chains) Cross-Adsorbed Secondary Antibody, Alexa Fluor 488, the latter only for p53 staining). Nuclei staining and image capture were performed as previously described^[22]^.

### Mitochondrial metabolism analysis

The measurements of oxygen consumption rates (OCR) in PLC/PRF/5 parental, PLC/PRF/5 EveR, PLC/PRF/5 SorR, and PLC/PRF/5 KO PER2 cells were performed by Seahorse XF analyzer (Seahorse Biosciences, North Billerica, MA, USA), using Cell Mito Stress Test kit (Seahorse Bioscience, 103015-100). The cells were seeded in Seahorse plates in a complete DMEM medium (1 × 10^4^ cells/well). Before Mito Stress analyses, the medium was replaced with a buffered base medium (Agilent Seahorse-103575-100, Agilent Technologies, Santa Clara, CA, USA) supplemented with 2 mM glutamine, 1 mM pyruvate, and 10 mM glucose at pH 7.4. The plates were equilibrated at 37 °C in a CO_2_-free incubator for at least 1 h. Basal OCR was determined in the presence of glucose, glutamine, and pyruvate. The measurement of the ATP production in the basal state was obtained from the decrease in respiration by inhibition of the ATP synthase with oligomycin (1.5 μm). Afterward, the mitochondrial electron transport chain was stimulated maximally by the addition of the uncoupler carbonylcyanide-p-trifluoromethoxyphenylhydrazone (2 µm). Spare respiratory capacity is the capacity of the cell to respond to an energetic demand and was calculated as the difference between maximal respiration and basal respiration. The non-mitochondrial respiration, due to a subset of cellular enzymes that continue to consume oxygen after the addition of rotenone/antimycin A (0.5 µm), was also calculated. The proton leak is indicated as the remaining basal respiration not coupled to ATP production and calculated as the difference between the minimum rate measurement after oligomycin injection and non-mitochondrial respiration. The mitochondrial respiration was expressed as the OCR per minute normalized to the number of cells. In our experimental conditions, the same cell number/well was plated before the OCR measurements; the cell count was obtained by using the Burker chamber.

### Cell proliferation assay

Parental PLC/PRF/5 and PLC/PRF/5 PER2 KO cells were used for proliferation assays. After trypsinization, 3 × 10^4^ PLC/PRF/5 parental cells were plated in 1 mL of complete culture medium in 24 well plates for 3 days. The plates were then placed in an incubator at 5% CO_2_ at 37 °C. After 24 h, PLC/PRF/5 parental cells were transfected with PER2 siRNA. Transfection was performed for 24 h using Lipofectamine 2,000, following the manufacturer’s protocol, with siRNA at a final concentration of 10 nm. Briefly, for each processed well, 1.05 μL siRNA and 1 μL Lipofectamine 2,000 were homogeneously dissolved in 50 μL siRNA transfection buffer Opti-MEM. After the next 24 h, PLC/PRF/5 parental cells were treated with EVE (10^-9^ M) and SOR (5 × 10^-6^ M) for 3 days in a complete culture medium. Concomitantly, PLC/PRF/5 PER2 KO cells (3 × 10^4^) were plated in 1 mL of complete culture medium in 24 well plates and, after 24 h, treated with EVE (10^-9^ M) and SOR (5 × 10^-6^ M) for 3 days in a complete culture medium. After 3 days of treatment, cells were harvested for DNA measurement. Measurement of total DNA content, representative of the number of cells, was performed using the bisbenzimide fluorescent dye (Hoechst 33258) (Boehring Diagnostic, La Jolla, CA), as previously described^[21]^.

### Migration assay

PLC/PRF/5 parental and PLC/PRF/5 PER2 KO cells were used for cell migration assay, evaluated by wound healing, based on the cell capability to cover a wound made by a tip in a well at a high cell density, as previously reported^[21]^.

PLC/PRF/5 Parental cells (8 × 10^4^) were seeded in 24 well plates and were grown until 100% of confluence. The cells were then transfected with siPER2 10 nm, using Lipofectamine 2,000 in the same condition described above, and after 24 h treated with EVE (10^-9^ M) and SOR (5 × 10^-6^ M), alone and in combination, in a culture medium with a lower percentage of serum (1%), to minimize cell proliferation but concomitantly to prevent apoptosis or cell detachment. Concomitantly, PLC/PRF/5 PER2 KO cells (8 × 10^4^) were plated in 1 mL of complete culture medium in 24 well plates and after 24 h treated with EVE (10^-9^ M) and SOR (5 × 10^-6^ M), in a culture medium with a lower percentage of serum (1%).

Scratch wounds were then visualized using an inverted microscope (Leica DMIL) at 5x magnification, and pictures were captured by a digital camera (Leica DFC420) after 24 h. The areas of the scratch-wounds were analyzed by Image J software and the closure was calculated by subtracting the remaining width of the scratch lines from the width of the baseline scratch (control). Each experiment was repeated at least three times. Four readings were made for each sample.

### Colony forming

Colony forming capability was evaluated in PLC/PRF/5 parental, PLC/PRF/5 EveR, PLC/PRF/5 SorR, and PLC/PRF/5 KO PER2 cells. For all cell models, 500 cells were seeded in six-well culture dishes and cultured in a complete medium. After 24 h adhesion, PLC/PRF/5 EveR and PLC/PRF/5 SorR cells were treated with EVE (10^-9^ M) and (SOR 5 × 10^-6^ M). Medium and compounds were refreshed every 3 days. After 21 days, formed colonies were stained and counted as previously reported^[19,23]^.

### Statistical analysis

Each proliferation and migration experiment was performed in quadruplicates and was replicated three times. Colony forming, WB and IF analysis were conducted as two independent experiments. Densitometry of IF protein signals was performed by assessing at least 6 different fields by two operators, which performed a single-blind evaluation. All statistical analyses were performed using GraphPad Prism 9 software. To evaluate a significant difference between the mean of more than two independent groups in the cell proliferation and cell migration assays, colony-forming and mitochondrial activity, the Analysis of Variance, followed by Tukey’s multiple comparative tests, was assessed. In colony-forming assay analysis, the non-parametric Mann-Whitney test was assessed to evaluate a significant difference between the mean of only two independent groups.

## RESULTS

### *In silico* analysis of PER2 expression in HCC cohort and cell lines

**Figure 1:**
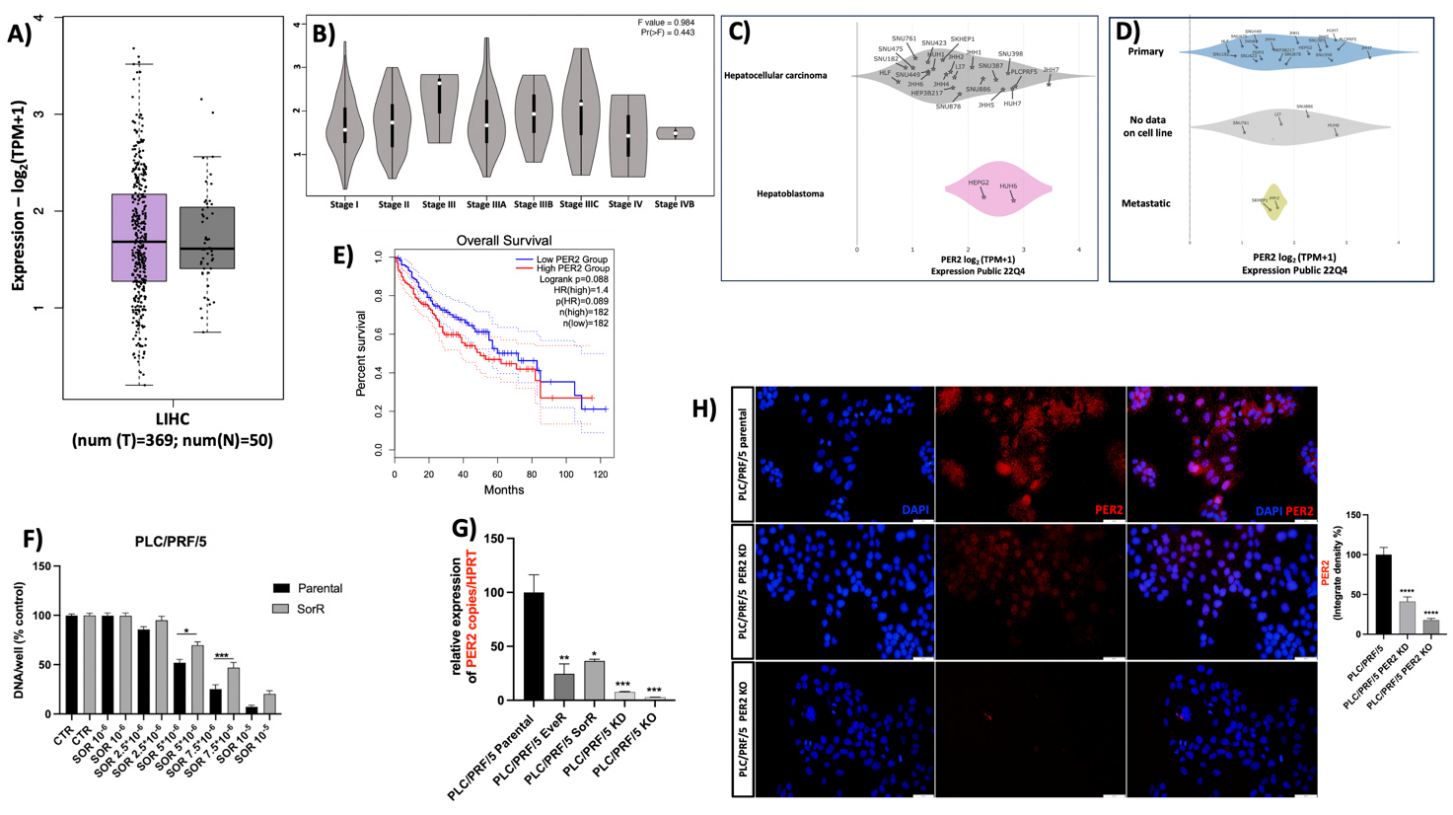
(A) *In silico* analysis of *PER2* mRNA expression in LIHC samples (purple boxplot) from TCGA cohort (obtained from RNAseq) compared with normal liver tissues (grey boxplot); (B) *In silico* analysis of *PER2* mRNA expression in different stages of LIHC; (C) and (D) *In silico* analysis of *PER2* mRNA expression in HCC cell lines from CCLE based on a dataset posted in 2022; (C) A comparison of *PER2* mRNA expression between HCC and hepatoblastoma cell lines; and (D) a comparison of *PER2* mRNA expression in HCC cell lines derived from human primary HCC, HCC cell lines with no clear origin, and HCC cell lines derived from human HCC metastasis; (E) Kaplan-Meier curve to assess the overall survival rate of patients with HCC depending on the PER2 gene expression; (F) SOR effect on cell line proliferation. Cell proliferation evaluated by DNA assay in PLC/PRF/5 cells: parental (black bars) and SorR (gray bars). The data presented are mean ± SEM of three independent experiments. *P < 0.05, ***P < 0.001 between groups; (G) *PER2* mRNA expression in PLC/PRF/5 parental, PLC/PRF/5 EveR, PLC/PRF/5 SorR, PLC/PRF/5 PER2 KD, and PLC/PRF/5 PER2 KO relative to HPRT housekeeping gene. *P < 0.05; **P < 0.01; ***P < 0.001; (H) PER2 protein expression in PLC/PRF/5 parental, PLC/PRF/5 PER2 KD, and PLC/PRF/5 PER2 KO with related densitometry. ****P < 0.0001. PER2: Period 2; LIHC: Liver Hepatocellular Carcinoma; CCLE: Cancer Cell Line Encyclopaedia; TCGA: The Cancer Genome Atlas; HCC: hepatocellular carcinoma; SOR: sorafenib; SEM: standard error of the mean; EveR: everolimus-resistant; SorR: sorafenib-resistant; KD: knockdown; KO: knockout; HPRT: Hypoxanthine Phosphoribosyltransferase 1.

The *in silico* analysis of *PER2* mRNA expression in 369 HCC samples and 50 normal liver tissue samples revealed no significative difference in *PER2* mRNA expression between tumor and normal tissues [Figure 1A], nor across HCC samples at different stages [Figure 1B] evaluated according to the American Joint Committee on Cancer (AJCC) TNM system.

However, when *PER2* mRNA expression was assessed across various human HCC cell lines divided into different groups, *in silico* analysis revealed that the PLC/PRF/5 cell is one of the human HCC cell lines with the highest expression of *PER2* [Figure 1C]. Interestingly, cell lines with metastatic origin, such as SKHEP1 and JHH2, displayed notably lower *PER2* mRNA levels [Figure 1D]. Survival analysis revealed that *PER2* is not a prognostic factor in patients with HCC [Figure 1E].

### PER2 expression in PLC/PRF/5 parental, PLC/PRF/5 EveR, PLC/PRF/5 SorR, PLC/PRF/5 PER2 KD and PLC/PRF/5 PER2 KO cell lines

The observation of lower *PER2* mRNA levels in cell lines of metastatic origin suggests that reduced *PER2* gene expression may contribute to the acquisition of aggressive phenotype and drug resistance in HCC. To further explore this hypothesis, *PER2* gene expression was assessed in PLC/PRF/5 parental, EveR, and SorR cell lines. The acquisition of SOR resistance in the PLC/PRF/5 SorR cell line was confirmed by evaluating cell proliferation [Figure 1F]. Consistent with the hypothesis, both PLC/PRF/5 EveR and SorR cells expressed significantly lower levels of *PER2* mRNA compared to the parental PLC/PRF/5 cells, with a reduction of 75.4% (*P* < 0.01) in PLC/PRF/5 EveR and a reduction of 63.6% (*P* < 0.05) in PLC/PRF/5 SorR cells [Figure 1G].

Following the observation of reduced *PER2* gene expression in drug-resistant cells, parental PLC/PRF/5 cells were manipulated to knock down or knock out the *PER2* gene. Functional assays were then performed to evaluate the potential effects of reduced *PER2* expression. Among the three tested siRNAs, hs.Ri.PER2.13.2 (siRNA 13.2) was selected for its superior efficacy in knocking down PER2 expression [Supplementary Figure 1]. Transfection of siRNA 13.2 and the PER2 CRISPR/Cas9 KO Plasmid (h2), designed to disrupt gene expression by inducing double-strand breaks in a 5’ constitutive exon of the human *PER2* gene, showed high efficacy in reducing *PER2* expression. Specifically, *PER2* mRNA levels were reduced by 92.3% in the PLC/PRF/5 PER2 KD cells (*P* < 0.001) and by 97.4% in PLC/PRF/5 PER2 KO cells (*P* < 0.001), compared to the parental PLC/PRF/5 cells [Figure 1G]. PER2 protein expression was further investigated by IF analysis in PLC/PRF/5 parental, PLC/PRF/5 PER2 KD, and PLC/PRF/5 PER2 KO cells. The results confirmed the silencing of *PER2* gene expression at the protein level: siRNA 13.2 strongly knocked down PER2 protein expression (59%, *P* < 0.0001), while PER2 CRISPR/Cas9 KO Plasmid almost completely knocked out PER2 expression (82.3%, *P* < 0.0001), as shown in Figure 1H.

### PER2 KD and KO induce an aggressive phenotype in the PLC/PRF/5 cell line

**Figure 2:**
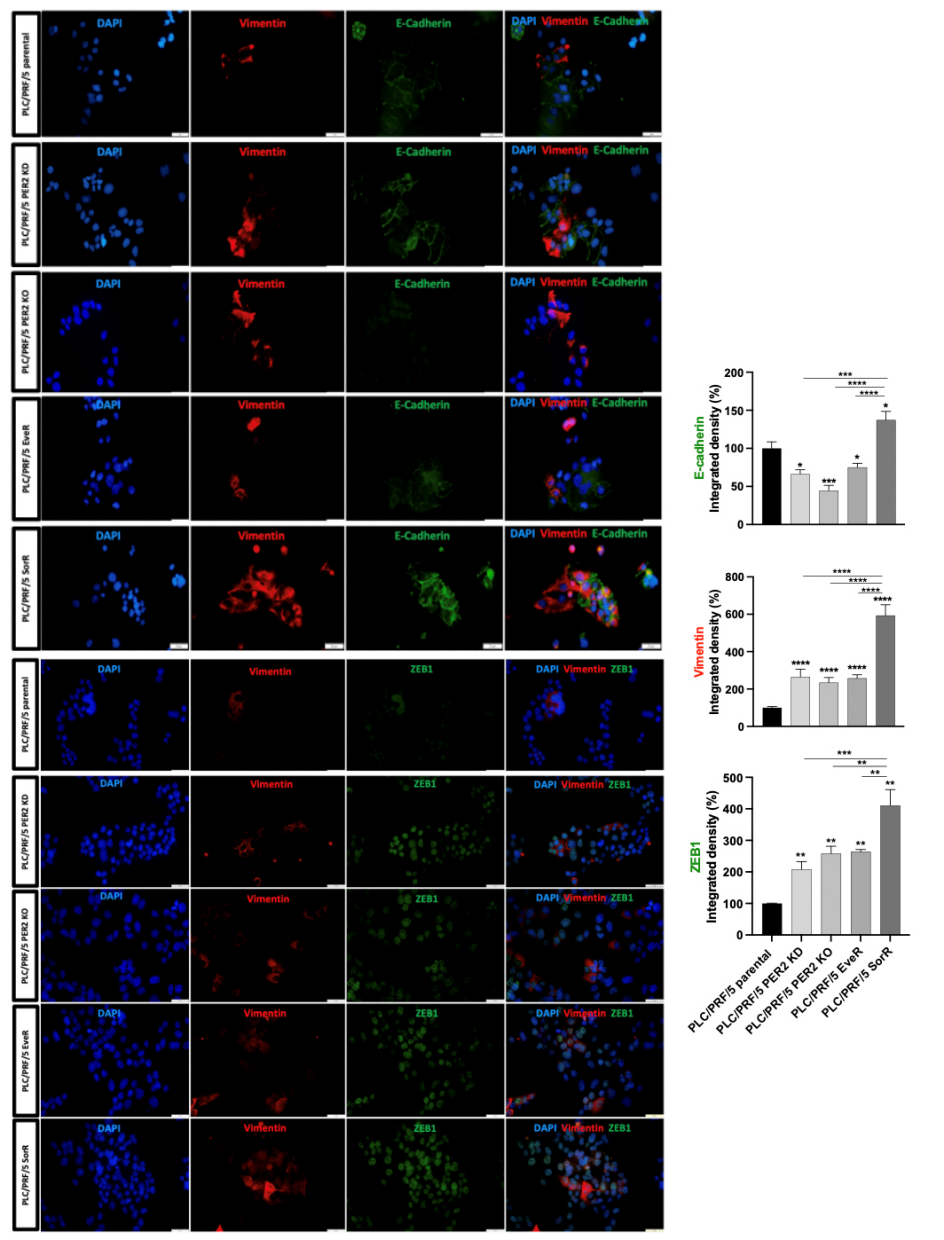
Evaluation of E-cadherin, vimentin and ZEB1 protein expression by immunofluorescent imaging in PLC/PRF/5 parental, PLC/PRF/5 PER2 KD, PLC/PRF/5 PER2 KO, PLC/PRF/5 EveR, and PLC/PRF/5 SorR cells. The data presented in the graphs are mean ± SEM of two independent experiments. *P < 0.05, ***P < 0.001, ****P < 0.0001 *vs.* control or among the groups. ZEB1: Zinc finger E-box binding homeobox 1; PER2: Period 2; KD: knockdown; KO: knockout; EveR: everolimus-resistant; SorR: sorafenib-resistant.

The protein expression levels of E-cadherin, vimentin, and ZEB1 were assessed to verify the involvement of the *PER2* gene in the acquisition of aggressive phenotype, through comparison between parental PLC/PRF/5 cells and the drug-resistant EveR and SorR PLC/PRF/5 cell lines. As confirmed in a previous study^[19]^, PLC/PRF/5 EveR cells showed a more aggressive phenotype compared to parental PLC/PRF/5 cells, with a significantly decreased expression of E-cadherin (24.9%, *P* = 0.04) and a significantly increased expression of vimentin (157.6%, *P* < 0.0001) and Zinc finger E-box binding homeobox 1 (ZEB1) (164.5%, *P* < 0.0022). Surprisingly, in PLC/PRF/5 SorR cells, significant increases in E-cadherin (37.2%, *P* = 0.03), vimentin (493.4%, *P* < 0.0001), and ZEB1 (310.5%, *P* < 0.0022) were observed, indicating an induction of the aggressive markers, vimentin and ZEB1, without a reduction in the epithelial marker, E-cadherin. Interestingly, the negative modulation of the *PER2* gene confirmed its involvement in the acquisition of the aggressive phenotype. Specifically, both *PER2* KD and KO led to a significant decrease in E-cadherin protein expression (33.8%, *P* = 0.04 for KD; and 55.5%, *P* = 0.0002 for KO) and a significant increase in vimentin and ZEB1 protein expression (PLC/PRF/5 PER2 KD: 166.0%, *P* < 0.0001 and 134.3%, *P* < 0.0001; PLC/PRF/5 PER2 KO: 108.2%, *P* = 0.0022 and 158%, *P* = 0.0022, respectively) [Figure 2].

### Loss of PER2 increases mitochondrial oxidative phosphorylation

**Figure 3:**
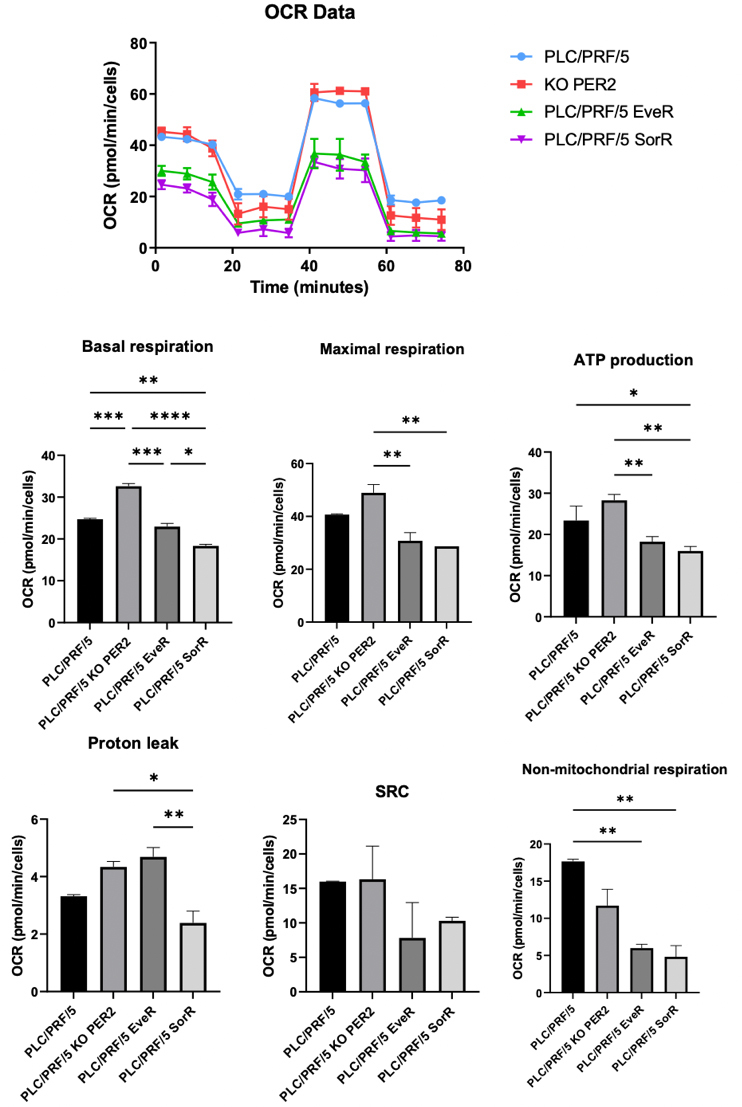
Representative graph of the Cell Mito Stress assay performed using the Seahorse XF analyzer. In the bar charts, each point in the OCR time courses represents the average of three technical replicates. Basal respiration, maximal respiration, proton leakage, ATP production, spare respiratory capacity, and non-mitochondrial respiration are reported. The values are expressed as mean ± SEM. *P < 0.05, **P < 0.01, ***P < 0.001, ****P < 0.0001 *vs.* control or among the groups. EveR: Everolimus-resistant; SorR: sorafenib-resistant; OCR: oxygen consumption rates; SEM: standard error of the mean.

It is conventionally assumed that cancer cell metabolism depends on lactate fermentation from glucose (aerobic glycolysis) irrespective of oxygen concentration, known as the Warburg effect^[24]^. However, strong evidence suggests that oxidative phosphorylation is not compromised in several cancers, but rather continues to contribute to energy production by cooperating with glycolysis to maintain cellular energetic balance^[25,26]^. Under physiological conditions, the circadian clock influences mitochondria dynamics and respiration^[27]^, and thus, the effects of PER2 KO on mitochondria morphology and function were investigated in the PLC/PRF/5 cell model and compared with parental, EveR, and SorR cells [Figure 3].

Mitochondrial metabolism was assessed by measuring OCR in PLC/PRF/5 parental, PLC/PRF/5 PER2 KO, PLC/PRF/5 EveR, and PLC/PRF/5 SoR cells. A significant increase in mitochondrial function was observed in PER2 KO cells, as evidenced by elevated basal and maximal OCR levels (32%, *P* = 0.007 and 20.25%, *P* = 0.27, respectively, compared to control) [Figure 3] and increased ATP production (20.9%, *P* = 0.10 *vs.* control) [Figure 3]. In contrast, chronic treatment with EVE or SOR, which is responsible for PER2 downregulation and aggressive phenotype acquisition, resulted in a reduced cellular oxidative capacity. Specifically, PLC/PRF/5 EveR and SoR cells showed reductions in basal (-7%, *P* = 0.34 EveR *vs.* control, -29.6%, *P* = 0.0001 EveR *vs.* PER2 KO, -25.6%, *P* = 0.0037 SorR *vs.* control, -43.7%, *P* < 0.0001 SorR *vs.* PER2 KO) and maximal respiration (-24.4%, *P* = 0.16 EveR *vs.* control, -37.1%, *P* = 0.009 EveR *vs.* PER2 KO, -29.6%, *P* = 0.012 SorR *vs.* control, -41.4% *P* = 0.0095 SorR *vs.* PER2 KO), as well as reduced ATP levels (-21.9%, *P* = 0.08 EveR *vs.* control, -35.4%, *P* = 0.0032 EveR *vs.* PER2 KO, -31.7%, *P* = 0.03 SorR *vs.* control, -43.5% *P* = 0.002 SorR vs. PER2 KO). This decrease was more evident in SorR cells, which also displayed the lowest proton leak values (-28.1%, *P* = 0.26 *vs.* control, -44.9%, *P* = 0.01 *vs.* PER2 KO, -49.1%, *P* = 0.005 *vs.* EveR) [Figure 3]. No significant difference was found in spare respiratory capacity between the different groups [Figure 3]. However, EveR and SorR cells exhibited a significant reduction in non-mitochondrial respiratory capacity compared to PLC/PRF/5 cells (-66,1%, *P* = 0.008; -72,6%, *P* = 0.0075, respectively) [Figure 3].

### Loss of PER2 expression induces resistance to EVE and SOR in HCC cell models

The aggressive phenotype that characterized the PLC/PRF/5 cell line with *PER2* KD or KO was also evident for the *in vitro* cell proliferation and migration and colony-forming response to EVE or SOR.

**Figure 4:**
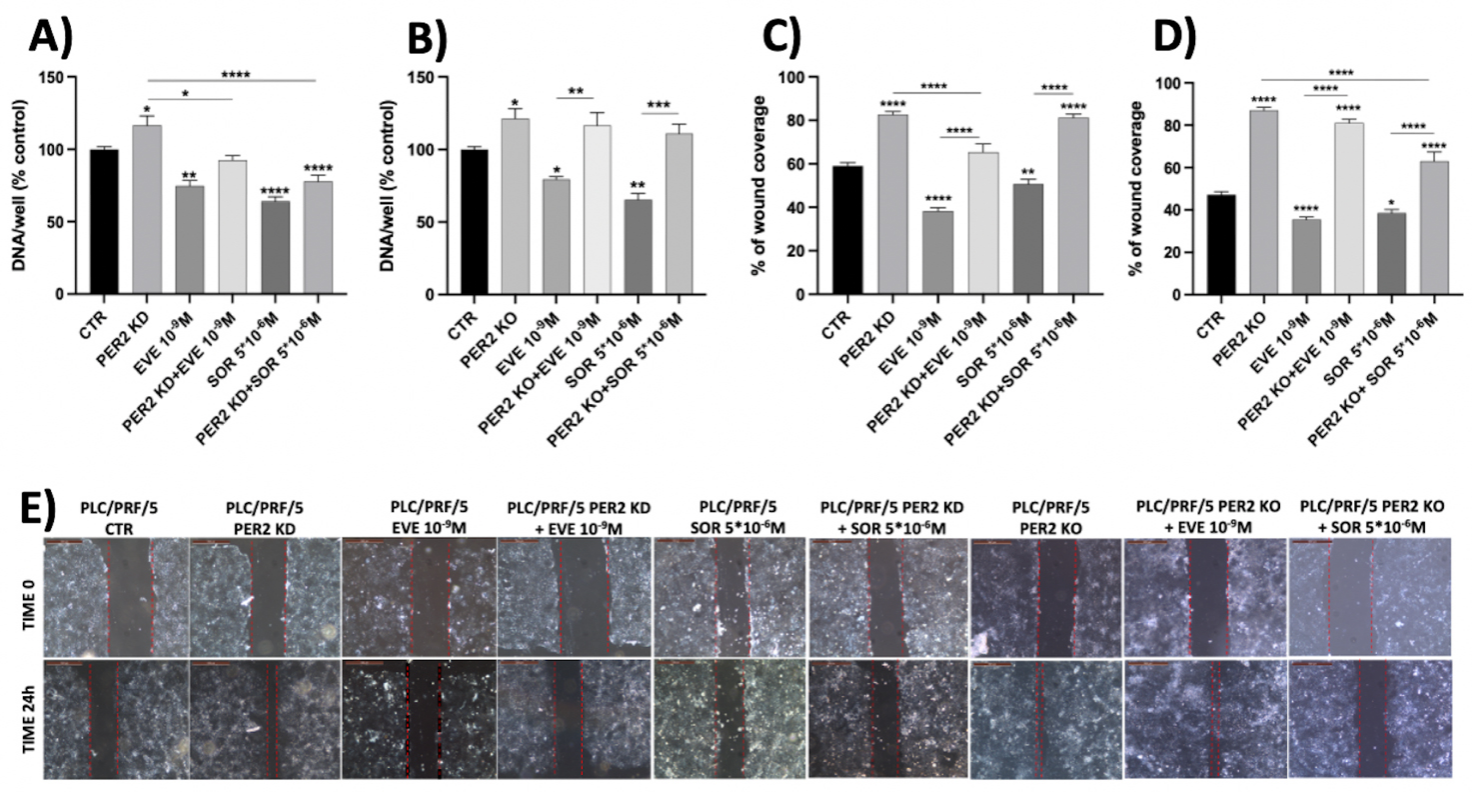
Cell proliferation evaluated by DNA assay in PLC/PRF/5 PER2 KD (A) and PLC/PRF/5 PER2 KO (B) with and without treatment with EVE (10^-9^ M) and (SOR 5 × 10^-6^ M). Cell migration evaluation by analysis of wound coverage in PLC/PRF/5 PER2 KD (C) and PLC/PRF/5 PER2 KO (D) and by scratch assay (E) with and without treatment with EVE (10^-9^ M) and SOR (5 × 10^-6^ M) compared to parental PLC/PRF/5. The data presented in the graphs are mean ± SEM of three independent experiments. *P < 0.05, **P < 0.01, ***P < 0.001, ****P < 0.0001 *vs.* control or among the groups. PER2: Period 2; KD: knockdown; KO: knockout; EVE: everolimus; SOR: sorafenib; SEM: standard error of the mean.

Treatment with EVE (10^-9^ M) induced a maximal significant inhibition of cell proliferation (25.37%, *P* < 0.01 *vs.* control) in the parental PLC/PRF/5 cell line. This effect persisted when *PER2* was knocked down by siRNA [Figure 4A], but was completely reverted when *PER2* was knocked out by plasmid (*P* < 0.01 *vs.* EVE 10^-9^ M) [Figure 4B]. The complete deletion of *PER2* gene also conferred resistance to SOR in the parental PLC/PRF/5 cell line; indeed, SOR (5 × 10^-6^ M) induced a maximal significant inhibition of cell proliferation by 35.9% (*P* < 0.0001 *vs.* control), an effect that remained following *PER2* KD by siRNA [Figure 4A] but was completely reversed by *PER2* KO by plasmid (*P* < 0.001 *vs.* SOR 5 × 10^-6^ M) [Figure 4B].

The aggressive phenotype was also observed in cell migration assays. Both *PER2* KD and KO significantly stimulated parental PLC/PRF/5 cell migration compared to the control (23.6%, *P* < 0.0001 and 39.8%, *P* < 0.0001, respectively) [Figure 4C-E]. On the contrary, EVE (10^-9^ M) and SOR (5 × 10^-6^ M) inhibited cell migration compared to the controls. Specifically, EVE (10^-9^ M) induced a maximal significant inhibition of cell migration (20.7%, *P* < 0.0001 *vs.* control) in the parental PLC/PRF/5 cell line, while this effect was completely reverted when *PER2* was knocked down by siRNA (*P* < 0.0001 *vs.* EVE 10^-9^ M) and when *PER2* was knocked out by plasmid (*P* < 0.0001 *vs.* EVE 10^-9^ M) [Figure 4C-E]. SOR (5 × 10^-6^ M) induced a maximal significant inhibition of cell migration (8.6%, *P* < 0.01 *vs.* control) in the parental PLC/PRF/5 cell line, while this effect was completely reverted when *PER2* was knocked down by siRNA (*P* < 0.0001 *vs.* SOR 5 × 10^-6^ M) and when *PER2* was knocked out by plasmid (*P* < 0.0001 *vs.* SOR 5 × 10^-6^ M) [Figure 4C-E].

**Figure 5:**
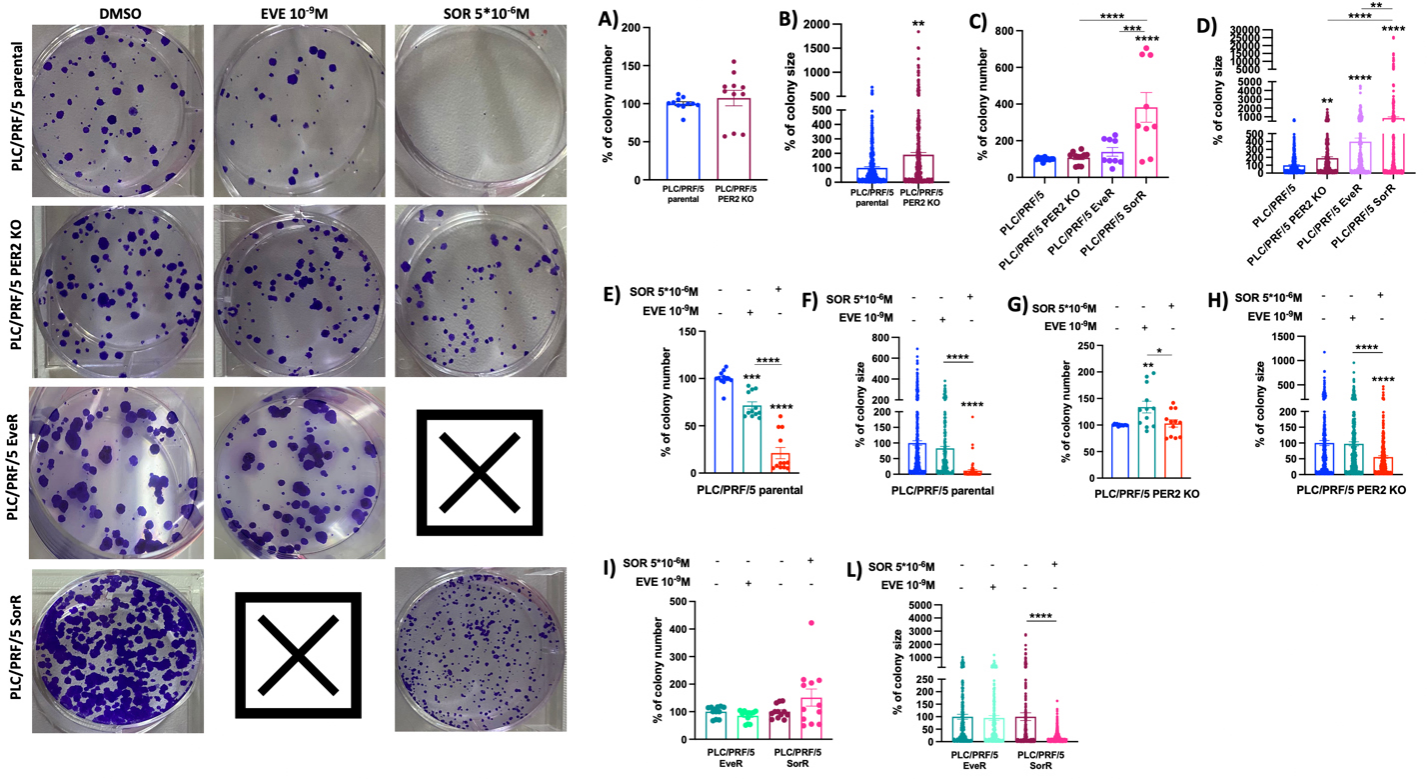
Images and statistical assessment of colony number (A, C, E, G and I) and size (B, D, F, H, and L) evaluated by colony formation assay in parental PLC/PRF/5, PLC/PRF/5 PER2 KO, PLC/PRF/5 EveR, and SorR with and without treatment with EVE (10^-9^ M) and SOR (5 × 10^-6^ M). The data presented in the graphs are mean ± SEM of two independent experiments. *P < 0.05, **P < 0.01, ***P < 0.001, ****P < 0.0001 *vs.* control or among the groups. PER2: Period 2; KO: knockout; EveR: everolimus-resistant; SorR: sorafenib-resistant; EVE: everolimus; SOR: sorafenib; SEM:standard error of the mean.

Furthermore, the lack of *PER2* expression, as well as the chronic drug treatment, conferred drug resistance by boosting colony-forming efficiency. While *PER2* KO and chronic EVE treatment did not confer greater capability to form colonies compared to parental PLC/PRF/5 cells, they significantly increased colony size (PLC/PRF/5 PER2 KO 90%, *P* = 0.0015 *vs.* parental PLC/PRF/5 and PLC/PRF/5 EveR 299%, *P* < 0.0001 *vs.* parental PLC/PRF/5). Chronic SOR treatment led to a significant increase in both colony number and size (PLC/PRF/5 SorR 282.1%, *P* < 0.0001 and PLC/PRF/5 SorR 755%, *P* < 0.0001 *vs.* parental PLC/PRF/5, respectively) [Figure 5A-D].

In parental PLC/PRF/5 cells, EVE (10^-9^ M) and SOR (5 × 10^-6^ M) significantly inhibited colony formation compared to untreated cells by inhibiting colony number (28.5%, *P* = 0.0001 and 78.8%, *P* < 0.0001, *vs.* PLC/PRF/5 cells, respectively) [Figure 5E] and colony size (SOR 5 × 10^-6^ M 88.6%, *P* < 0.0001 *vs.* PLC/PRF/5 cells) [Figure 5F], whereas *PER2* KO prevented EVE (10^-9^ M) and SOR (5 × 10^-6^ M) efficiency [Figure 5G and H], as well as EVE and SOR chronic treatment [Figure 5I and L].

### Subcellular localization of PER2 characterizes HCC cell models resistant to EVE and SOR

**Figure 6:**
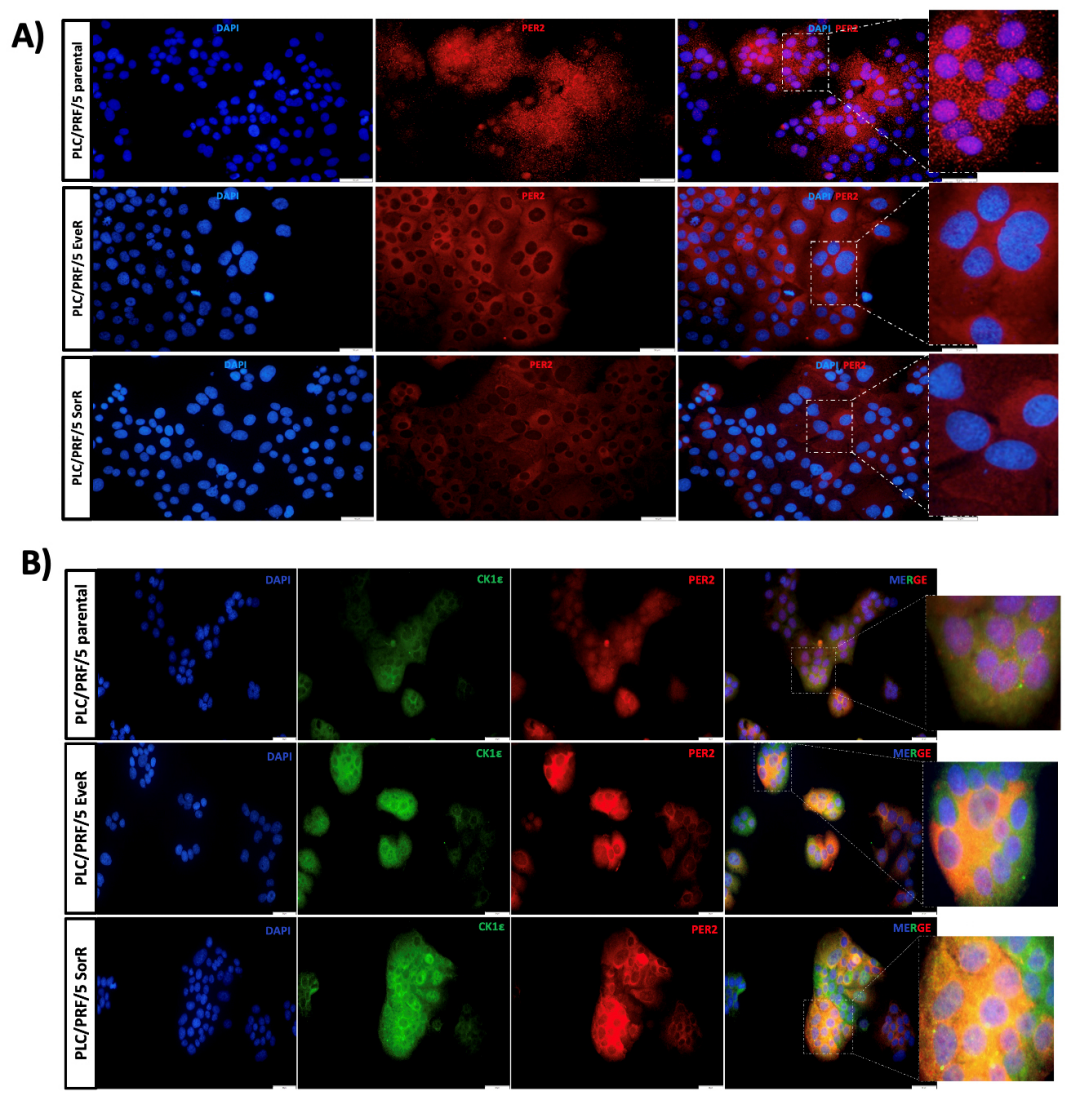
Immunofluorescent evaluation of (A) PER2 protein expression in PLC/PRF/5 parental, PLC/PRF/5 EveR, and PLC/PRF/5 SorR, and (B) colocalization of PER2 and CK1ε in PLC/PRF/5 parental, PLC/PRF/5 EveR, and PLC/PRF/5 SorR. PER2: Period 2; CK1ε: casein kinase 1ε; EveR: everolimus-resistant; SorR: sorafenib-resistant.

**Figure 7:**
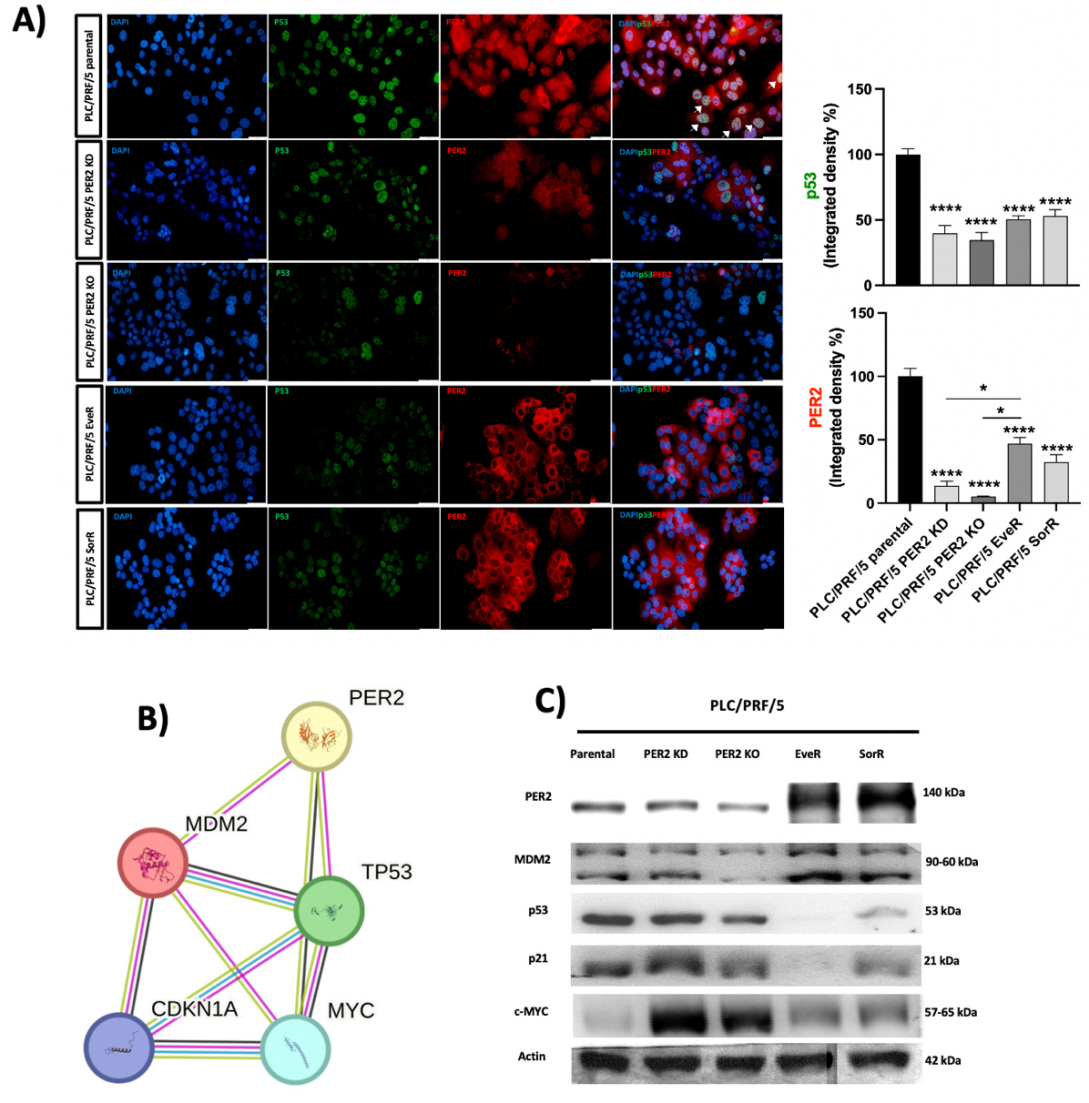
(A) PER2 and p53 protein expression in PLC/PRF/5 parental, PLC/PRF/5 PER2 KD, PLC/PRF/5 PER2 KO, PLC/PRF/5 EveR, and PLC/PRF/5 SorR cells. White arrows indicate the co-expression of PER2 and p53 proteins. The data presented in the graphs are mean ± SEM of two independent experiments. *P < 0.05, ****P < 0.0001; (B) Protein-Protein Interaction Networks Functional Enrichment Analysis using STRING software to analyze and visualize the network connection among PER2, MDM2, p53, p21, and c-MYC; (C) Western blot analysis of PER2, MDM2, p53, p21, and c-MYC parental PLC/PRF/5, PLC/PRF/5 PER2 KD, PLC/PRF/5 PER2 KO, PLC/PRF/5 EveR, and PLC/PRF/5 SorR cells. PER2: Period 2; KD: knockdown; KO: knockout; EveR: everolimus-resistant; SorR: sorafenib-resistant; SEM: standard error of the mean; MDM2: mouse double minute 2 homolog; c-MYC: cellular myelocytomatosis oncogene.

Although PER2 expression was significantly reduced in PLC/PRF/5 EveR and SorR cells compared to parental PLC/PRF/5 cells, it remained detectable [Figure 1E]. This observation prompted further investigation into comparing the patterns of PER2 protein expression across these three cell models. Interestingly, IF staining showed that in PLC/PRF/5 parental cells, PER2 was localized to both the nucleus and cytoplasm, displaying a distinct punctate staining pattern [Figure 6A]. In contrast, PER2 staining in PLC/PRF/5 EveR and SorR cells was predominantly cytoplasmic, more diffuse, and less intense, suggesting a loss of PER2 nuclear translocation in drug-resistant HCC models [Figure 6A]. These findings were corroborated by WB analysis [Supplementary Figure 2]. Studies performed on mammalian cells indicate that CK1ε may regulate the subcellular localization of PER2 via phosphorylation, potentially promoting PER2 degradation through the proteasomal pathway^[28,29]^. Therefore, to elucidate the mechanism underlying the cytoplasmic retention of PER2 in resistant cells, the potential colocalization of CK1ε and PER2 was evaluated by IF in PLC/PRF/5 parental, EveR, and SorR cells. In PLC/PRF/5 parental cells, PER2 was mainly localized in the nucleus, whereas CK1ε was predominantly found in the cytoplasm. Conversely, in PLC/PRF/5 EveR and SorR cells, PER2 was essentially expressed in the cytoplasm and colocalized with CK1ε [Figure 6B]. Beyond localization, quantitation of the integrated density of the signal obtained by IF revealed that PLC/PRF/5 parental cells expressed higher levels of PER2 compared to PLC/PRF/5 PER2 KD (-86.26%), PLC/PRF/5 PER2 KO (-94.74%), PLC/PRF/5 EveR (-53.07%), and SorR cells (-67.56%) (*P* < 0.0001) [Figure 7A].

As PER2 in the nucleus may bind to the tumor suppressor protein p53, a key regulatory checkpoint component of cell cycle progression and the cellular response to genotoxic stress, the expression and localization of p53 were also evaluated in PLC/PRF/5 parental, PLC/PRF/5 PER2 KD, PLC/PRF/5 PER2 KO, PLC/PRF/5 EveR, and SorR cells. Parental PLC/PRF/5 cells showed significantly higher nuclear p53 expression compared to PLC/PRF/5 PER2 KD (-60.36%), PLC/PRF/5 PER2 KO (-65.40%), PLC/PRF/5 EveR (-47.03%), and SorR cells (-45.20%) (*P* < 0.0001) [Figure 7A]. In PLC/PRF/5 parental cells, p53 colocalized with PER2 (white arrows in Figure 7A). However, in PLC/PRF/5 PER2 KD and PLC/PRF/5 PER2 KO cells, the reduction of PER2 nuclear localization was accompanied by a reduction of p53 levels in the nucleus, affecting their nuclear colocalization [Figure 7A]. Interestingly, in PLC/PRF/5 EveR and SorR cells, nuclear p53 levels were significantly reduced compared to PLC/PRF/5 parental cells, and PER2-p53 interaction was absent due to cytoplasmic localization of PER2 [Figure 7A].

### Expression and subcellular localization of PER2 modulate the expression of repressors and oncogenes involved in key cancer-related pathways

*In silico* analysis by the STRING tool revealed that PER2 interacts with the repressors, p53 and p21, and the oncogenes, MDM2 and c-MYC [Figure 7]. These interactions were further confirmed using far WB [Figure 7C]. KD and, more notably, KO of PER2 reduced expression of p53, p21, and MDM2, while c-MYC expression was increased. Interestingly, PLC/PRF/5 EveR and SorR cells, which predominantly exhibit PER2 in the cytoplasm, displayed higher expression levels of MDM2 and c-MYC, but significantly lower or nearly absent levels of p53 and p21, compared to PLC/PRF/5 parental cells [Figure 7C].

## DISCUSSION

A subset of patients with advanced HCC do not achieve long-term clinical benefits from systemic therapies due to acquired drug resistance mediated by several mechanisms. Combined with the rising incidence of HCC, this resistance contributes to listing HCC as a highly fatal disease^[30]^. Investigating the complex and diverse molecular mechanisms adopted by tumor cells to bypass drug efficiency may enable the identification of new therapeutic targets and strategies aimed at improving patient outcomes.

The findings of the current study demonstrate that the lack of PER2 expression is associated with an aggressive phenotype and acquired drug resistance in HCC cell models, which involves regulation of epithelial-mesenchymal transition (EMT) and changes in mitochondrial plasticity. Moreover, these results revealed that HCC cell models of drug resistance, obtained with chronic exposure to EVE and SOR to acquire an aggressive phenotype, are associated with a peculiar cytoplasmic localization of PER2. This deficit in PER2 localization was accompanied by a marked reduction in the tumor suppressor p53 and its major target p21, along with increased levels of the oncogenes MDM2 and c-MYC.

The mammalian *Period* gene family encodes three homologous proteins, known as PER1, PER2, and PER3. PER2 is well known to exert a crucial role in controlling cell proliferation by regulating the expression of several downstream genes, including tumor protein p53 (TP53) and MYC. Moreover, aberrant expression of PER2 may lead to malignant cell transformation by inducing alteration in cell cycle progression and checkpoint responses to DNA damage^[31]^. Consistently, downregulation of the clock gene PER2 has been linked to the development of a variety of cancers. Therefore, PER2 has been hypothesized to function as a tumor suppressor in the liver, acting from hepatocarcinogenesis initiation to progression.

In the current study, *in silico* computational analysis was conducted to compare the expression of the *PER2* gene using the Genotype-Tissue Expression (GTEx) data on 369 HCC samples and 50 normal liver tissue from TCGA. No significant difference in *PER2* gene expression was observed between the two groups. In addition, the Kaplan-Meier curve indicated no prognostic role of *PER2* in HCC [Figure 1E]. However, several studies have reported that *PER2* mRNA expression is significantly decreased in HCC tissues compared to paired noncancerous tissues^[7,14-18]^, supporting our hypothesis.

The results from the current study demonstrated that, among the HCC cell lines, cell models derived from metastatic lesions exhibited lower *PER2* mRNA expression compared to cell lines derived from primary tumors, thus raising the hypothesis that reduced *PER2* expression could have a causal role in the acquisition of metastatic phenotype, characterized by gained mesenchymal features, uncontrolled cellular growth capacity, and drug resistance. To test this hypothesis, the PLC/PRF/5 human cell line, parentally expressing the highest *PER2* mRNA levels among the cell lines examined, was used. Moreover, chronic treatment of PLC/PRF/5 cells with EVE^[19]^ and SOR was useful in generating drug-resistant cells (EveR and SorR cells). Finally, genetic manipulation of PLC/PRF/5 cells allowed the development of PER2 KD and KO cell lines (PLC/PRF/5 PER2 KD and KO, respectively). Interestingly, *PER2* mRNA expression was reduced in drug-resistant cells (PLC/PRF/5 EveR and SorR), and PER2 protein was predominantly localized in the cytoplasmic cell compartment in these cells, in contrast to its distribution in the parental PLC/PRF/5 cells. Both the reduced mRNA expression of PER2 and its altered localization in the cytoplasm were associated with an aggressive phenotype and upregulation of EMT protein markers such as vimentin and ZEB1 proteins in EveR and SorR cells, as well as both the PER2 KD and KO in PLC/PRF/5 cells. These results suggest that not only the expression levels but also the PER2 cellular localization are critical in the acquisition of aggressiveness in the HCC cell model. These results are consistent with previous findings in oral squamous cell carcinoma, where cytoplasmic localization of PER2 was associated with increased expression of mesenchymal markers^[32]^. Interestingly, in PLC/PRF/5 SorR cells, an upregulation of both vimentin and E-cadherin was observed. E-cadherin is a cell-cell junction protein and a typical marker of epithelial cells that plays an important function in epithelial cell adhesion and tissue architecture preservation. Loss of E-Cadherin during the EMT promotes cancer cell migration, invasion, and metastasis^[33]^. Nevertheless, elevated E-cadherin protein levels have also been associated with unfavorable clinical features in epithelial tumors^[34]^, mainly due to its ability to activate pro-oncogenic signaling pathways such as Wnt/*β*-catenin and to interact with various growth factor receptors^[33]^. Therefore, we could speculate that the E-cadherin expression rebound in PLC/PRF/5 SorR could reflect a cancer cell dedifferentiation that leads to tumor progression.

Intriguingly, bioinformatic investigation of PER2-related differentially expressed genes (DEG) in human HCC samples revealed their enrichment in gene categories related to mitochondrial oxidative phosphorylation, respiratory electron transport, translation elongation, and other related pathways^[16]^.

The acquired aggressiveness of PLC/PRF/5 PER2 KO cells was supported by the preservation of mitochondrial function, as indicated by the analyses of oxidative capacities. It is conceivable that there has been an increase in de-novo synthesis of mitochondria to meet metabolic and energy demands^[35]^. Chronic treatment with EVE, but especially with SOR, appeared to have the most impactful effect on mitochondrial function and, over time, can induce drug resistance in cancer cells by triggering adaptive changes in mitochondrial processes. Indeed, PLC/PRF/5 SorR cells showed the lowest proton leak values, a mechanism that leads to high coupling efficiency between substrate oxidation and oxidative phosphorylation. This, in turn, increases the mitochondrial membrane potential beyond the critical threshold of reactive oxygen species (ROS) production^[36]^. While ROS can be toxic and induce cell death, cancer cells may, over time, develop adaptive mechanisms to manage and neutralize ROS (e.g., increased expression of antioxidant proteins), thereby enabling survival despite ongoing mitochondrial damage^[37]^.

EVE is an mTOR inhibitor, and mTOR plays a critical role in regulating cell growth, metabolism, and survival^[38]^. Chronic mTOR inhibition may promote mitochondrial biogenesis by increasing the expression of proteins involved in mitochondrial replication. Although this may compensate for mitochondria damage in the short term, the resulting increase in mitochondrial mass may still be dysfunctional over time, complicating treatment outcomes^[39]^. Lastly, a downward trend in spare respiratory capacity was observed following treatments with EVE or SOR, indicating the inability of these cells to respond to an increased energy demand or stress. Additionally, PLC/PRF/5 EveR or SorR cells showed a significant reduction in non-mitochondrial respiratory capacity, likely as a consequence of their metabolic reprogramming and altered ATP utilization, reflecting the broader adaptive mechanisms that tumor cells undergo to survive under chronic treatment conditions.

The acquired aggressiveness was further supported by the elevated rate of cell proliferation, migration, and colony formation in PLC/PRF/5 PER2 KD and PLC/PRF/5 PER2 KO cells, as previously demonstrated for PLC/PRF/5 EveR cells^[19]^. Notably, PLC/PRF/5 PER2 KD and PLC/PRF/5 PER2 KO cells acquired resistance to the antiproliferative effect of EVE and SOR, suggesting that reduced or absent PER2 expression, and its altered cellular localization, as observed in EveR and SorR cells, play a key role in the acquisition of drug resistance in HCC cell model.

CK1ε is a member of the serine/threonine protein kinases family, which is known to phosphorylate a broad range of substrates^[28,29]^. Although CK1ε does not show circadian rhythm, it regulates members of the molecular clock, including the PER clock genes, and primarily controls the duration of the repressor state. At the end of the circadian day, CK1ε binds to and phosphorylates PER1, inducing a conformational change that hinders its nuclear localization signal. This inhibits the nuclear entry of PER1 and leads to its cytoplasmic accumulation and subsequent degradation. Likewise, CK1ε phosphorylates PER2 and primes it for ubiquitination and degradation^[28,29]^. Our observation of complete cytoplasmic localization of PER2 led us to hypothesize that PER2 binding to CK1ε controls its subcellular localization and, potentially, its protein turnover. Results from the current study demonstrate that acquired drug resistance was associated with PER2 and CK1ε colocalization in the cytoplasm, which may facilitate PER2 ubiquitination and degradation, resulting in reduced expression levels.

The clock gene PER2 also binds to the tumor suppressor protein p53, a key regulatory checkpoint component, whose modulation regulates cell cycle arrest, DNA repair, apoptosis, or senescence in response to genotoxic stress, depending on the activation of specific p53 target genes^[40]^. Additionally, p53 can act as a transcriptional repressor of several genes, including c-fos, myc, and VEGF-A, thereby modulating cell proliferation, survival, and angiogenesis^[41-43]^. PER2 binding to p53 modulates its stability, cellular localization, and transcriptional activity^[40]^. Thus, inactivation or loss of p53 leads to drug resistance through the suppression of apoptotic pathways. In agreement with a positive correlation between PER2 and TP53 reported by Chen *et al.* in a cohort of 80 HCC samples^[16]^, we observed that PER2 protein expression is positively associated with p53 protein levels. In PLC/PRF/5 PER2 KD and PLC/PRF/5 PER2 KO cells, the lack of nuclear PER2 expression was associated with the reduction in p53 expression. Similarly, cytoplasmic PER2 protein expression in the PLC/PRF/5 EveR and SorR cells was associated with reduced p53 nuclear expression, confirming that the lack of nuclear PER2 expression negatively influences p53’s stability. Consequent to reduced p53 expression, decreased levels of p21 were observed in PLC/PRF/5 PER2 KO, PLC/PRF/5 EveR, and PLC/PRF/5 SorR cells. Loss of p21 expression has been considered a negative predictor for response to systemic adjuvant therapies, and is considered a key factor in mediating drug resistance in human cancers^[44]^.

Several pieces of evidence suggest that p53 function is inhibited by elevated levels of MDM2. MDM2 binds to p53, acting as a p53 ubiquitin ligase that targets p53 for degradation, thereby downregulating the tumor-suppressive p53 pathways^[45]^. Therefore, MDM2 might be considered the most important negative regulator of p53. Additionally, MDM2 overexpression correlates with metastasis and advanced forms of several cancers, including colon, breast, and prostate cancers, and MDM2 overexpression is often associated with drug resistance^[46,47]^. Remarkably, the results of the present study showed that the cytoplasmic PER2 localization was associated with increased MDM2 overexpression, further confirming MDM2 as a marker of tumor aggressiveness and drug resistance. This observation may also have important implications in the context of immunotherapy, which has emerged as the first-line treatment for advanced HCC^[9]^. Elevated MDM2 expression can impair tumor cell killing by T cells, thus promoting immune evasion^[48]^.

Besides TP53, PER2 is known to regulate numerous cell cycle genes, including c-MYC^[49]^. c-MYC influences cell cycle progression and plays an important role in supporting the changes triggering cellular transformation. Its activation controls cellular proliferation, growth, and metabolism by conferring growth advantage and promoting tumorigenesis. Moreover, c-MYC upregulation confers mesenchymal features in several cancers, including HCC. We previously demonstrated that c-MYC protein levels are elevated in PLC/PRF/5 EveR cells compared with parental cells^[19]^. In the current study, we demonstrated that PER2 KD or KO also increased c-MYC expression, consistent with observations in PLC/PRF/5 EveR and SorR cells, where PER2 was localized to the cytoplasm.

Despite the promising results of the current study supporting the tumor-suppressive role of PER2 in HCC, the main limitation of this study is that it was conducted in a single HCC cell model. Nevertheless, previous studies have highlighted the involvement of PER2 in HCC development, although its role in targeted drug resistance has not been previously explored.

In conclusion, the current study demonstrates that chronic treatment with EVE and SOR may reduce PER2 expression and promote its cytoplasmic localization, where PER2 colocalizes with CK1ε that facilitates its proteasomal degradation. This altered expression of PER2 triggers the upregulation of several mesenchymal markers, such as vimentin and ZEB1, and oncogenes, such as c-MYC and MDM2, along with the downregulation of p53 and p21. The rearrangement in gene expression is associated with the acquisition of aggressiveness and drug resistance to target therapy in an HCC cell model. Further studies are warranted to investigate the impact of PER2 expression on the efficacy of immunotherapy in HCC, which could contribute to improved patient management and treatment outcomes.

## DECLARATIONS

### Availability of data and materials

The datasets used and/or analyzed during the current study are available from the corresponding author upon reasonable request.

### Copyright

© The Author(s) 2025.
