# Supplementary material for: PER2 expression and cellular localization play a critical role in tumor aggressiveness and drug resistance in an in vitro model of hepatocellular carcinoma
Source: Cancer Drug Resist. 2025 Jun 3;8:26. doi: 10.20517/cdr.2024.193 (PMC12162184; doi:10.20517/cdr.2024.193)
Supplement: Supplementary file 1 [file cdr-8-26-SupplementaryMaterials.pdf]

PER2 expression and cellular localization play a critical role in tumor aggressiveness and drug resistance in an *in vitro* model of hepatocellular carcinoma

Mariarosaria Negri[1,2,#], Feliciana Amatrudo[1,2,#], Donatella Paola Provvisiero[2], Roberta Patalano[2], Giovanna Trinchese[3], Fabiano Cimmino[3], Cristina de Angelis[2], Chiara Simeoli[2], Renata Simona Auriemma[2], Maria Pina Mollica[3], Annamaria Colao[2,4], Rosario Pivonello[2,4], Claudia Pivonello[5]

[1]Department of Wellness, Nutrition and Sport, Pegaso Telematic University, Naples 80143, Italy.

[2]Department of Clinical Medicine and Surgery, Section of Endocrinology, Diabetology, Andrology and Nutrition, Federico II University, Naples 80131, Italy.

[3]Department of Biology, Federico II University, Naples 80126, Italy.

[4]UNESCO Chair for Health Education and Sustainable Development, Federico II University, Naples 80131, Italy.

[5]Department of Public Health, Federico II University, Naples 80131, Italy.

[#]These authors have contributed equally to this work.

Correspondence to: Prof. Claudia Pivonello,Department of Public Health, Federico II University, Via Sergio Pansini, 5, Naples 80131, Italy, E-mail: claudia.pivonello@unina.it

ORCID: Claudia Pivonello (0000-0003-4276-8600)

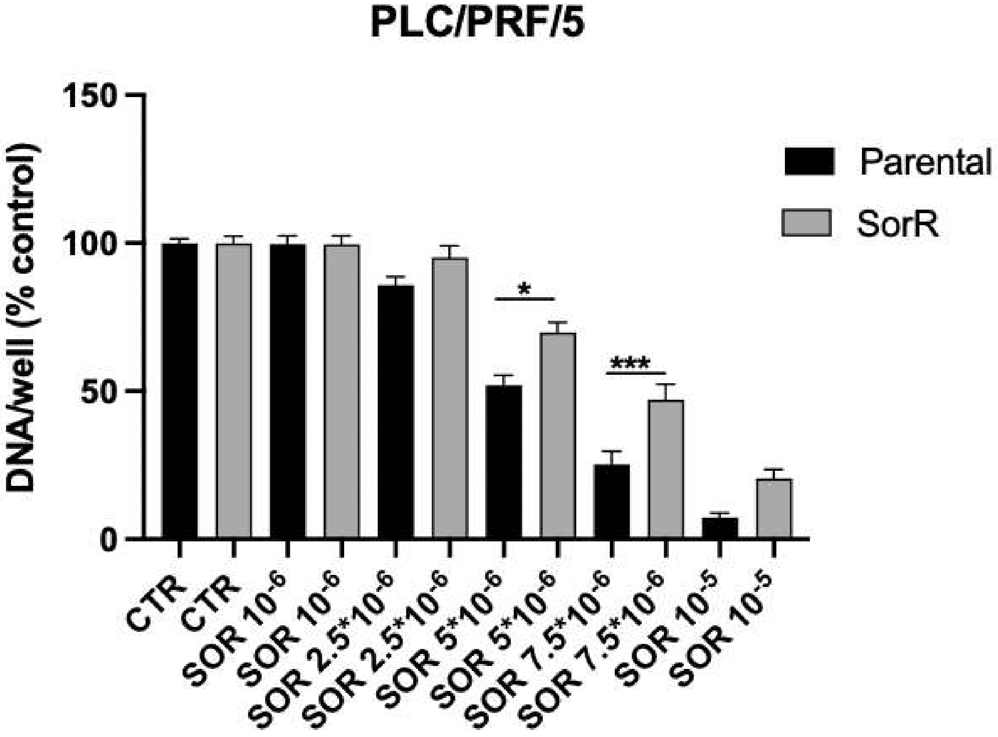

Supplementary Figure 1

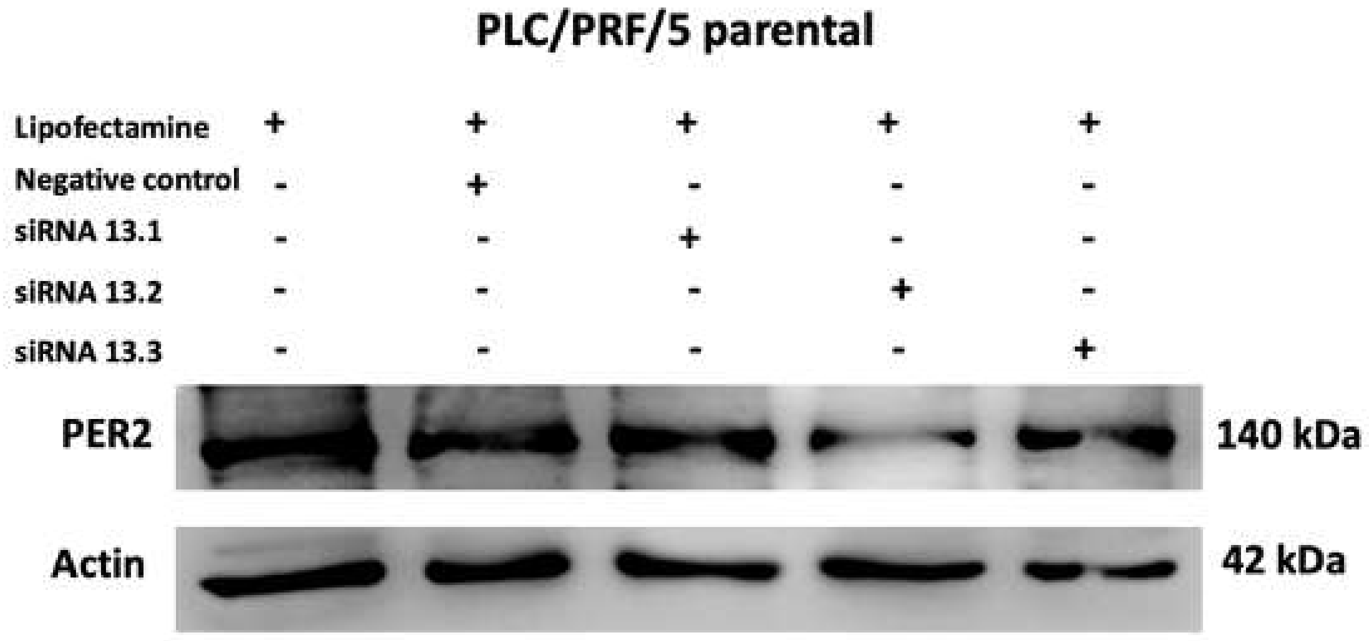

Supplementary Figure 2
